# Senator Backus’ Report on Petitions for Homœopathic College

**Published:** 1846-05-01

**Authors:** 


*Report of Committee on Medical Societies and Medical Colleges, on petitions for a Homœopathic College.*—We have here another report from Dr. Backus, and his colleagues of the committee, which, if our journal had a larger proportion of non-medical readers, than it has, we should publish entire. The committee deem it proper to state what Homœopathy is—i. e. what are its peculiar tenets. They seem to think that this only is necessary for the community to form an opinion as to its merits. There is no doubt that a large portion of those even who favor the system, do not really know the length and breadth of its absurdity; and it would therefore be well for the public if the above short report were to be pretty extensively diffused by the newspaper press. The committee, each member of which (if we mistake not) is a medical man, respond most heartily to the desire for a Homœopathic college, and reported a bill accordingly!

